# Radiomics analysis for prediction and classification of submucosal tumors based on gastrointestinal endoscopic ultrasonography

**DOI:** 10.1002/deo2.374

**Published:** 2024-05-07

**Authors:** Hui Zhou, Guoliang Wei, Junke Wu

**Affiliations:** ^1^ College of Science University of Shanghai for Science and Technology Shanghai China; ^2^ Business School University of Shanghai for Science and Technology Shanghai China

**Keywords:** endoscopic ultrasound, extra trees, radiomics, submucosal tumor, tumor classification

## Abstract

**Objectives:**

To identify and classify submucosal tumors by building and validating a radiomics model with gastrointestinal endoscopic ultrasonography (EUS) images.

**Methods:**

A total of 144 patients diagnosed with submucosal tumors through gastrointestinal EUS were collected between January 2019 and October 2020. There are 1952 radiomic features extracted from each patient's EUS images. The statistical test and the customized least absolute shrinkage and selection operator regression were used for feature selection. Subsequently, an extremely randomized trees algorithm was utilized to construct a robust radiomics classification model specifically tailored for gastrointestinal EUS images. The performance of the model was measured by evaluating the area under the receiver operating characteristic curve.

**Results:**

The radiomics model comprised 30 selected features that showed good discrimination performance in the validation cohorts. During validation, the area under the receiver operating characteristic curve was calculated as 0.9203 and the mean value after 10‐fold cross‐validation was 0.9260, indicating excellent stability and calibration. These results confirm the clinical utility of the model.

**Conclusions:**

Utilizing the dataset provided curated from gastrointestinal EUS examinations at our collaborating hospital, we have developed a well‐performing radiomics model. It can be used for personalized and non‐invasive prediction of the type of submucosal tumors, providing physicians with aid for early treatment and management of tumor progression.

## INTRODUCTION

With the advancement of endoscopic ultrasonography (EUS) technology, the detection rates of submucosal tumors (SMTs) within the gastrointestinal tract have significantly increased.^1^ These tumors, emerging from the pathological transformation of non‐epithelial mesenchymal tissues within the gastric wall, represent a diverse group of neoplastic lesions. They include gastric submucosal leiomyoma (GSL), neuroendocrine tumors (NETs), gastric ectopic pancreas (GEP), gastrointestinal stromal tumors (GIST), gastric mucosa lipoma (GML), and hemangiomas, among others, with GSL and GIST being notably prevalent.^2^ The detection of SMTs often occurs incidentally during gastroscopy or is prompted by clinical symptoms related to the tumor's size and location, such as palpable abdominal masses, gastrointestinal bleeding, or abdominal pain. The European Society of Gastrointestinal Endoscopy guidelines from 2022 highlight the significance of early diagnosis in enhancing treatment outcomes by outlining indications for SMT treatment based on malignancy risk, symptomatic presentation, and considerations for patients undergoing bariatric surgery.^3^, ^4^

Despite the benefits, traditional endoscopy primarily offers a superficial examination, limited in its capacity to visualize the deeper mucosal layers. Consequently, biopsy samples obtained during such procedures might not yield sufficient pathological information for an accurate determination of the tumor's nature, especially given the challenge of distinguishing between benign and malignant lesions.^5^ EUS emerges as a superior modality by integrating the benefits of ultrasound and endoscopy, enabling detailed visualization of SMTs' external morphology and probing their origin layers, which is crucial for accurate pathology assessment and prognosis estimation.^6^, ^7^ Additionally, EUS provides invaluable insights into membrane integrity and other prognostic factors, thereby predicting surgical outcomes and enhancing patient management.^8^ This study leverages EUS by concurrently acquiring white light images (WLI) and ultrasound images, playing a pivotal role in the precise identification of tumor types.

The digital age has led to an unprecedented gathering of patient and tumor data for research. Radiomics, a key player in medical imaging, uses sophisticated algorithms to identify numerous features from tumor images.^9^, ^10^, ^11^ It transforms EUS images into detailed data, revealing patterns in tumor traits that traditional imaging might miss.^12^, ^13^ As a revolutionary technique, radiomics converts images into detailed features, indicating pathological and physiological conditions.^14^ ^,15^ Employing advanced algorithms, advances precision medicine, though challenges remain in representing SMTs with traditional features in our dataset. This study introduces wavelet and Laplacian of Gaussian (LoG) filters for better feature visibility, demonstrating their importance in classifying tumor types.^16^While traditional diagnostic methods like EUS‐guided fine‐needle aspiration and unroofing biopsy provide direct tissue analysis, radiomics offers a non‐invasive alternative, analyzing the entire lesion with potential for higher diagnostic accuracy and complementing these methods by identifying patterns not visible through conventional techniques.

We introduce a radiomics model underpinned by customized least absolute shrinkage and selection operator (MyLASSO) regression and the extremely randomized trees (Extra Trees) algorithm. Utilizing EUS‐obtained ultrasound images as model inputs, we adopted the area under the receiver operating characteristic curve (AUC) as the evaluation criterion. Our objective was to assess the ultrasound radiomics model's feasibility in accurately predicting various tumor types and identifying key features to ensure model stability and precision.

## MATERIALS AND METHODS

### Patients

**FIGURE 1:**
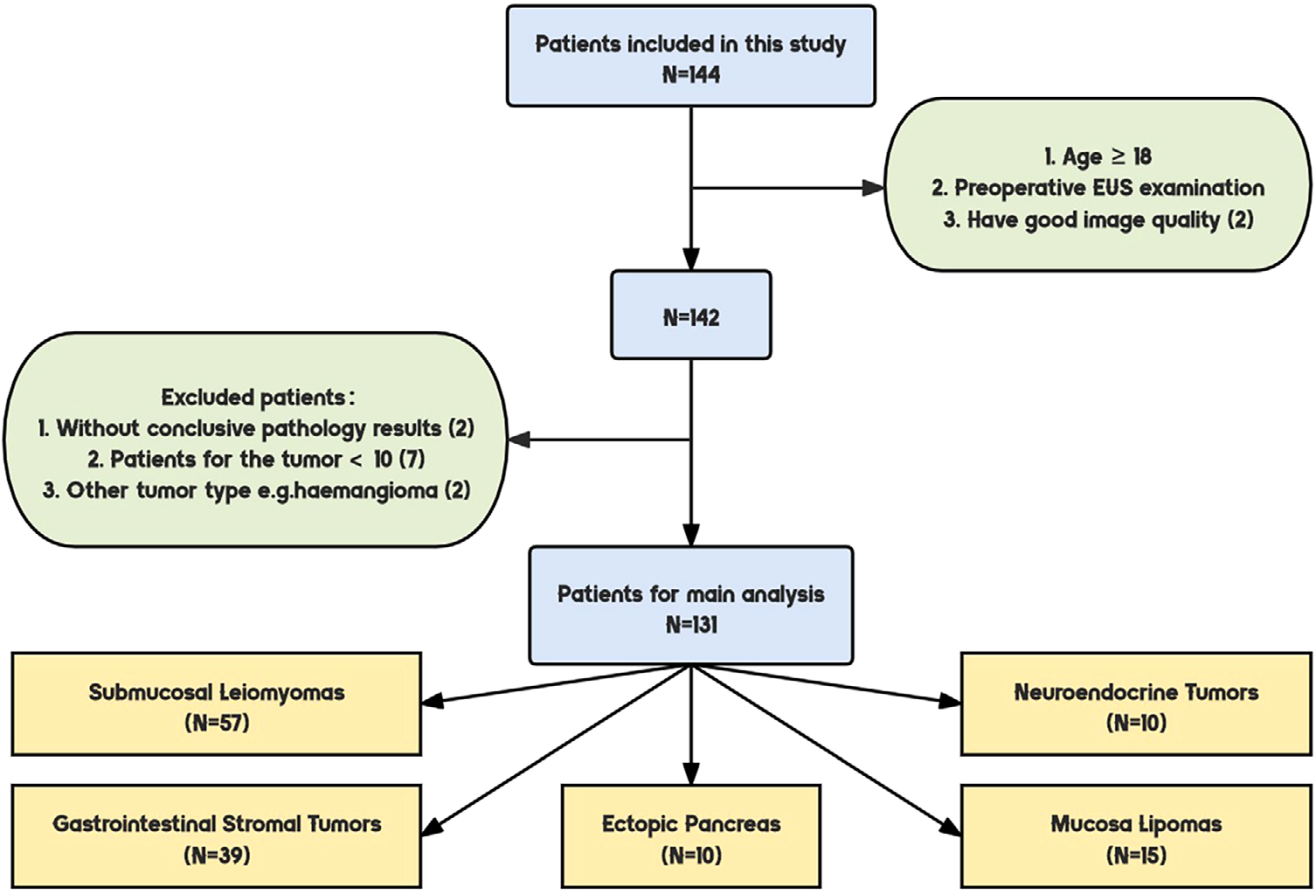
Flowchart for the exclusion of patients: Patients who are minors and whose pictures are not clear are first excluded; Patients who have inconclusive pathology results; The tumor has fewer than 10 patients or the tumor is not a submucosal tumor; for example, hemangioma. The numbers in brackets correspond to the number of cases excluded for each condition.

The data was obtained from the Gastrointestinal Endoscopy Center of Shanghai Sixth People's Hospital, affiliated with Shanghai Jiaotong University. It included 2042 images from 144 patients, gathered between January 2019 and October 2020. It spans various age groups and genders, ensuring a broad representation. The tumor cases included 57 GSLs, 39 GISTs, 10 NETs, 10 GEPs, and 15 GMLs. Thirteen atypical cases were omitted from the analysis due to limited representation, inadequate sample size, or other factors (Figure 1). All patients provided informed consent, and the study adhered to the ethical guidelines of the 1975 Declaration of Helsinki (6th revision, 2008), as confirmed by prior institutional committee approval. All patients underwent multimodal ultrasonography, including WLI and EUS. The final diagnoses for all patients were based on histopathological results from biopsies, including both surgical and endoscopic biopsies.

**FIGURE 2:**
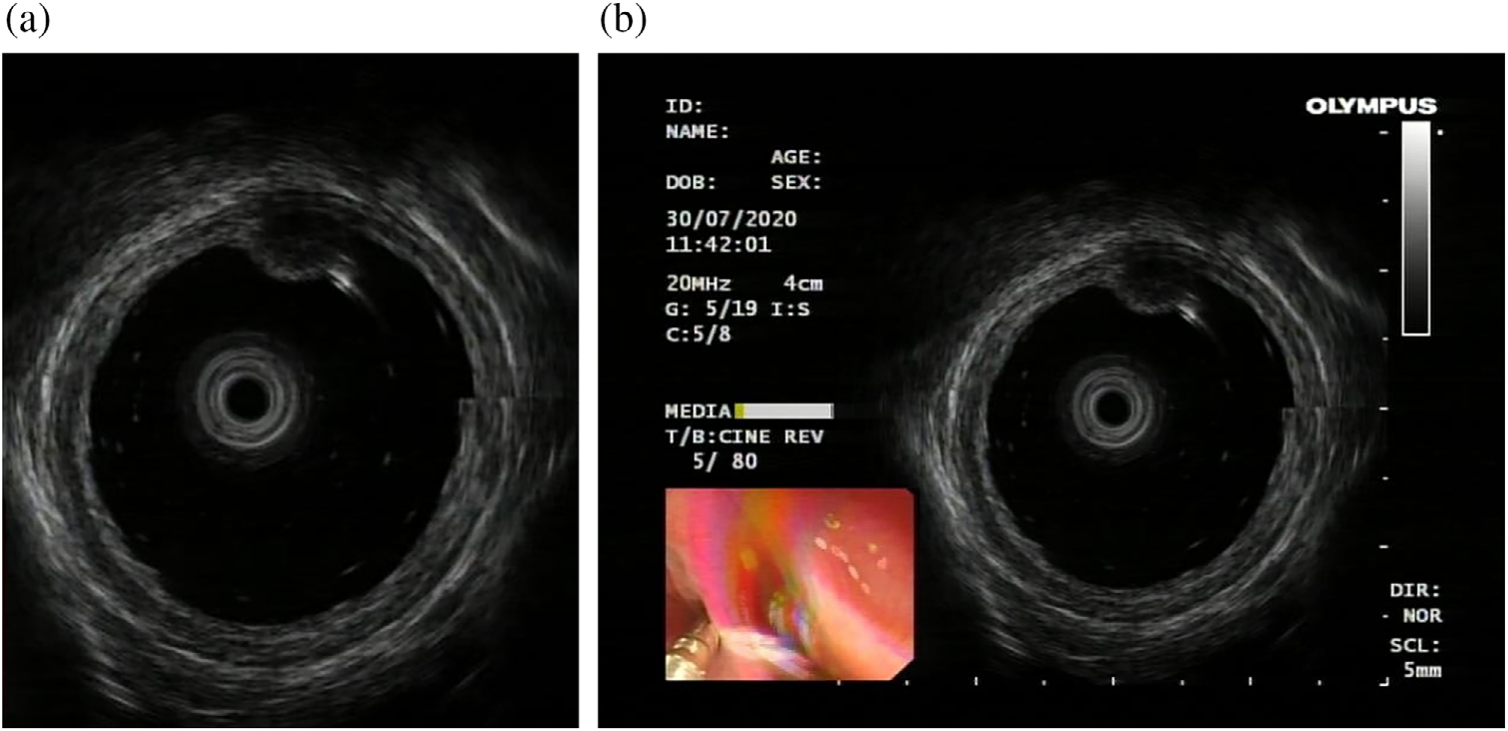
The data set: (a) The pixel size is 764 × 572, including dates, machine parameters, and so on. (b) The pixel size is 388 × 457, without irrelevant information.

The data encompasses patients' age, gender, and lesion location. The initial dataset, with a resolution of 764 × 572 pixels, contained extraneous details like dates, machine parameters, and corresponding WLI. These images were cropped to 388 × 457 pixels (Figure 2). In this set, all ultrasound images were processed with wavelet and LoG filters to minimize ultrasound noise before feature extraction. Tumor location annotations and region of interest (ROI) segmentation were manually executed by skilled ultrasound doctors.

Inclusion criteria were as follows: (1) age ≥18 years; (2) confirmed diagnosis of SMTs (including GSLs, GISTs, NETs, GEPs, and GMLs); (3) preoperative EUS examination; (4) exclusion of patients with indeterminate tumor types in the dataset. More details are shown in Figure 1. In total, 131 patients were included in our study. The patients were randomly divided into a training group (*n* = 105) and a testing group (*n* = 26) in an 8:2 ratio. The clinical characteristics of all patients are shown in Table 1. The baseline characteristics of patients in the training and test cohorts are shown in Table 2. There were no statistically significant differences between the two cohorts.

**TABLE 1:** Clinical and histologic characteristics of the study population.

	GSL (*n* = 57)	GIST (*n* = 39)	NET (*n* = 10)	GEP (*n* = 10)	GML (*n* = 15)	*p*‐value
Age (years)*^a^*	55.54 ± 10.153	59.41 ± 10.197	49.80 ± 14.688	44.10± 16.347	58.67 ± 9.271	>0.05
Gender						>0.05
Male	19 (33.3%)	19 (48.7%)	7 (70%)	4 (40%)	6 (40%)	
Female	38 (66.7%)	20 (51.3%)	3 (30%)	6 (60%)	9 (60%)	
Dominant lesion size*^b^*	0.764 ± 0.677	1.553 ± 1.738	0.557 ± 0.027	1.92 ± 0.85	2.68 ± 1.912	>0.01*^c^*
Uniformity of echo						<0.01
Uniformity	52	16	6	1	14	
Non‐uniformity	5	23	4	9	1	
Tumor location						
Esophagus	31	0	0	0	1	
Fundus	16	31	0	0	1	
Stomach	10	8	0	1	3	
Duodenum	0	0	1	1	0	
Rectum	0	0	9	0	0	
Gastric sinus	0	0	0	8	8	
Colon	0	0	0	0	2	

^a,b^Data are mean ± standard deviations.

^c^The *p*‐value between GSL and GEP was 0.004, but not analytical.

Abbreviations: GEP, gastric ectopic pancreas; GIST, gastrointestinal stromal tumor; GML, gastric mucosa lipoma; GSL, gastric submucosal leiomyoma; NET, neuroendocrine tumor.

**TABLE 2:** Baseline characteristics of patients in the training and test cohort.

	Age (years)	Gender	Male	Female	Dominant lesion size*^b^*
GSL (*n* = 46) Training	55.19 ± 10.497		15 (32.6%)	31 (67.4%)	0.831 ± 0.623
GSL (*n* = 11) Test	57 ± 8.854		4 (36.4%)	7 (63.6%)	0.713 ± 0.598
***p*‐value**	**0.601**	**0.483**			**0.839**
GIST (*n* = 31) Training	60.322 ± 9.914		15 (48.4%)	16 (51.6%)	1.479 ± 1.694
GIST (*n* = 8) Test	55.875 ± 11.192		4 (50%)	4 (50%)	1.683 ± 1.833
***p*‐value**	**0.277**	**0.442**			**0.934**
NET (*n* = 8) Training	53.5 ± 15.128		6 (75%)	2 (25%)	0.586 ± 0.021
NET (*n* = 2) Test	49 ± 11.314		1 (50%)	1 (50%)	0.524 ± 0.032
***p*‐value**	**0.352**	**0.401**			**0.441**
GEP (*n* = 8) Training	44.375 ± 16.698		3 (37.5%)	5 (62.5%)	1.88 ± 0.792
GEP (*n* = 2) Test	43 ± 21.213		1 (50%)	1 (50%)	1.962 ± 0.878
***p*‐value**	**0.922**	**0.398**			**0.121**
GML (*n* = 12) Training	59.75 ± 9.687		5 (41.7%)	7 (58.3%)	2.59 ± 1.736
GML (*n* = 3) Test	54.33 ± 7.094		1 (33.3%)	2 (66.7%)	2.87 ± 2.023
***p*‐value**	**0.385**	**0.476**			**0.478**

Abbreviations: GEP, gastric ectopic pancreas; GIST, gastrointestinal stromal tumor; GML, gastric mucosa lipoma; GSL, gastric submucosal leiomyoma; NET, neuroendocrine tumor.

### Pathologic assessment of response

All lesion annotations were performed by more than three experienced pathologists who conducted histopathological examination and analysis to determine the tumor type. The final pathology results were then reviewed by dedicated gastrointestinal pathologists for further assessment, ensuring accurate pathological findings.

### Tumor masking

**FIGURE 3:**
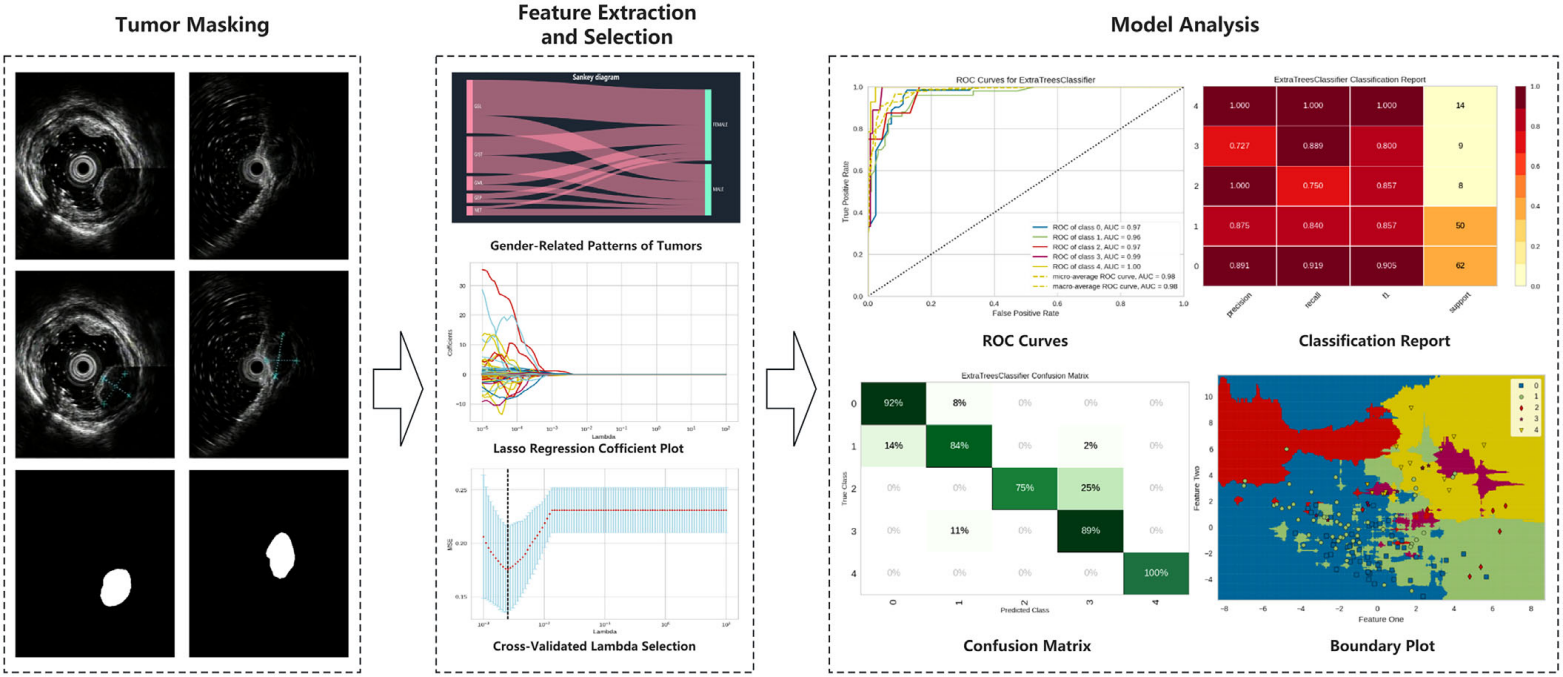
The workflow of the radiomics model construction. First, the dataset was labeled with ROIs after manual segmentation by the doctors; a total of 1952 features were extracted from the images that had been subjected to wavelet filters and LoG filters. Secondly, the Sankey diagram, statistical test, and MyLASSO were applied to the feature selection. Finally, the radiomics prediction model was constructed based on the selected features by Extra Trees. Abbreviations: MSE, mean square error; ROC, receiver operating characteristic; ROI, regions of interest; LoG, Laplacian of Gaussian; MyLASSO, the customized least absolute shrinkage and selection operator.

The patient's EUS images (including WLI) were analyzed by two radiologists (Dr. Li, a radiologist with 10 years of experience in tumor imaging, and Dr. Xia, a physician with 7 years of experience in tumor imaging). Both performed manual creation of regions of interest on the images under masking of histopathological findings (Figure 3), including the entire tumor except the bowel lumen.

### Radiomic feature extraction and statistical analysis

All EUS images were taken with OLYMPUS EU‐ME3 at frequencies of 7.5 and 12 MHz. Clinical features included patient age, gender, tumor size, and so on (Table 1). We extracted 1952 features from each EUS image: 216 original, 496 from wavelet decomposition, and 1240 from LoG filters. Radiomic features included shape, first‐order, and texture features, which comprised five categories: gray‐level dependence matrix (GLDM), gray‐level co‐occurrence matrix (GLCM), gray‐level run length matrix (GLRLM), gray‐level size zone matrix (GLSZM) and neighboring gray‐tone difference matrix (NGTDM). All of the radiomic features were extracted from three different image types (one original and two derived images) except the shape features. Wavelet features were obtained through wavelet decomposition, which can capture the high‐frequency information of the images and serve as an important component of image features. The LoG features combined texture information displaying various frequency scales and different feature orientations within the tumor volume.^17^ These features have been widely used in previous radiomics studies.^18^, ^19^

Additionally, we categorized SMTs into five types: GSL (Label = 0), GIST (Label = 1), NET (Label = 2), GEP (Label = 3), and GML (Label = 4), and performed necessary statistical analysis. After removing defective data, we used Pearson's chi‐square test to compare tumor type differences based on attributes like age and gender. Statistical analyses were done with IBM SPSS Statistics 26.0, with significance levels at 0.05 or 0.01.

### Radiomics model construction

The radiomics signature workflow involves lesion segmentation, feature extraction, selection, and model analysis (Figure 3). The model's efficacy was assessed using various machine learning models, with the Extra Trees model finalizing the tumor type classifications. This algorithm, a variation of Random Forest, selects suboptimal attributes. This reduces computational time, increases tree diversity, and offers strong generalization and noise resilience.

The best λ value that maximized the AUC of the subjects in the dataset was selected as the best regularisation parameter. After the choice of λ value, the radiomic score for each patient in the validation dataset was calculated using an extreme random forest model. Accuracy, AUC, sensitivity, and specificity values were calculated as metrics to assess the quantitative discrimination performance of radiomic features in this dataset (Table 3).

**TABLE 3:** Ten‐fold cross‐validation of Extra Trees (full features and selected features).

	Accuracy		AUC		Sensitivity		Specificity	
Fold	Full	Selected	Full	Selected	Full	Selected	Full	Selected
0	0.8235	0.7941	0.9548	0.9397	0.8235	0.7941	0.8537	0.8348
1	0.7353	0.7647	0.9349	0.9585	0.7353	0.7647	0.6825	0.7051
2	0.7273	0.7576	0.9293	0.9136	0.7273	0.7576	0.7446	0.7748
3	0.7879	0.8182	0.9301	0.9268	0.7879	0.8182	0.8273	0.8275
4	0.7273	0.6970	0.8480	0.8795	0.7273	0.6970	0.7455	0.6713
5	0.7879	0.8182	0.9109	0.9200	0.7879	0.8182	0.7441	0.8263
6	0.6364	0.6870	0.8350	0.8820	0.6364	0.6870	0.6320	0.6992
7	0.7879	0.8485	0.8863	0.9488	0.7879	0.8485	0.7966	0.8538
8	0.7879	0.8485	0.9650	0.9704	0.7879	0.8485	0.7413	0.8057
9	0.7879	0.7273	0.9283	0.9213	0.7879	0.7273	0.7414	0.7100
Mean	0.7589	0.7771	0.9123	0.9260	0.7589	0.7771	0.7509	0.7708
SD	0.0509	0.0545	0.0409	0.0284	0.0509	0.0545	0.0613	0.0645

Abbreviation: SD, standard deviation.

### Feature selection method

To minimize overfitting in the final model, we first excluded features without significant tumor type differences. We then applied the customized LASSO algorithm, which sets coefficients of less significant features to zero. The model (MyLASSO) is defined as follows:

$$\;y_{i}=\sum_{j=1}^{n}\alpha_{j}x_{ij}+\alpha_{0}+\beta \;$$

For patients with GSL $y_{i}=\;0$; for patients with GIST $y_{i}=\;1$; for patients with NET $y_{i}=\;2$; for patients with GEP $y_{i}=\;3$; for patients with GML $y_{i}=\;4$; n is the total number of features used in the model; $x_{ij}\left(j\;=\;1,2,\text{…},n\right)$ is the different features for each case, i is the number of cases; $\alpha_{j}\left(j\;=\;0,1,2,\text{…},n\right)$ is the parameter of the model, and β is the error term.

The MyLASSO algorithm uses regularization to pare down parameter values, simplifying the model while keeping key features. Its objective function has a data fitting term (minimizing squared residuals) and a penalty term (penalizing model parameter absolute values). This penalty increases with the regularization parameter λ, making the model focus on fewer crucial features as λ rises.

Its optimization problem can be expressed as minimizing the following cost function:

$$\sum_{i\;=\;1}^{N}{\left[y_{i}-S\left(\sum_{j\;=\;1}^{n}\alpha_{j}x_{ij}+\alpha_{0}\right)\right]}^{2}+\lambda \sum_{j\;=\;1}^{n}\left|\alpha_{j}\right|$$

where $y_{i}$ is the outcome for patient i, N is the number of patients, S is the sigmoid function, $x_{ij}$ is the $j_{th}$ feature of the $i_{th}$ patient and λ is the regularization parameter. The sigmoid function S is defined as follows:

$$S\;=\frac{1}{e^{x}}\;$$

The MyLASSO penalty $\sum_{j\;=\;1}^{n}\left|\alpha_{j}\right|$ was applied in order to set some parameters $\alpha_{j}$ to zero, which can generate a sparse model. Finally, the algorithm selected features with greater contributions.

## RESULTS

### Clinical characteristics

Table 1 lists patients' clinical details. With an average age of 55.81 ± 11.689 years, 40.85% were male, and 58.45% were female. Figure 3’s Sankey diagrams depict tumor type distribution by gender. Continuous variables were analyzed using mean ± SD and t‐tests; categorical ones used chi‐square tests. Statistical analysis results for the training and test groups, including age, gender, tumor size, and their *p*‐values, are shown in Table 2. Results revealed no significant age or gender differences across tumor types, deeming them non‐influential in tumor classification.

Most tumors predominantly affected females, except for NET due to fewer cases. All intergroup *p*‐values were above 0.05, suggesting no notable associations. Although tumor sizes varied little, GSL and NET were typically smaller, while GIST, GEP, and GML were larger. Different tumors had distinct ultrasound echoes, emphasizing in‐depth ultrasound image feature analysis.

### Model selection

The dataset consists of 475 lesion images, with 428 for model training and 47 for evaluation. The model utilized all 1952 extracted features from each image and assessed performance using the AUC value. A higher AUC value is deemed superior to accuracy in Radiomics.^20^ After examination, the Extra Trees Classifier achieved the highest AUC value of 0.8998, making it the final classification model.

**FIGURE 4:**
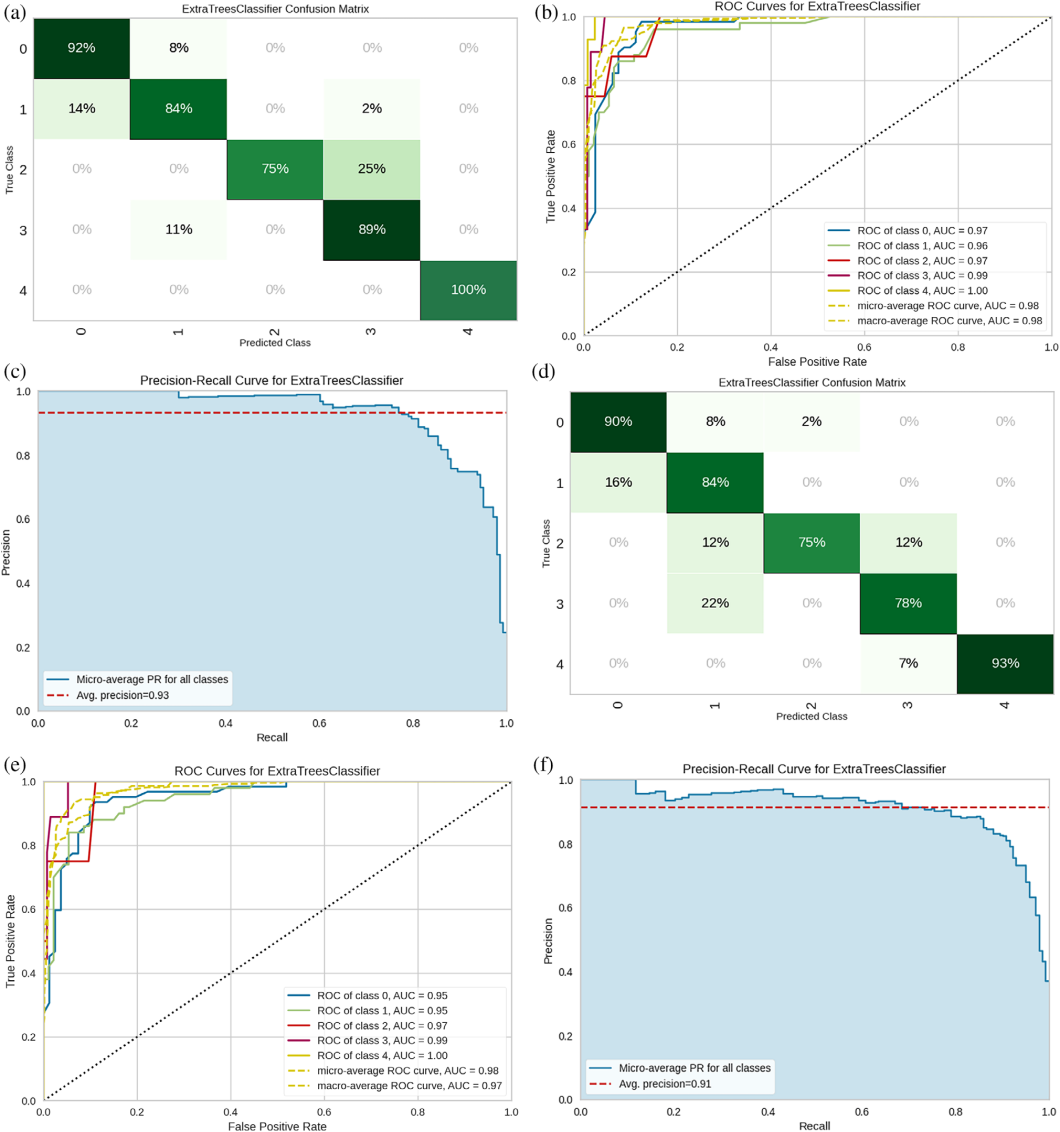
Results of classification predictions by the customized Radiomics model: (a) Results of selected features; (b) ROC of the model (selected features); (c) PR curve of the model (selected features); (d) Results of full features; (e) ROC of the model (full features); (f) PR curve of the model (full features). Abbreviations: ROC, the receiver operating characteristic curve; PR curve, the precision‐recall curve.

To further validate the stability of the Extra Trees model, 10‐fold cross‐validation was performed on the dataset. This approach helps reduce the impact of random variations and detect and mitigate the risk of overfitting. It helps to capture the performance of the model on the entire dataset and provides an objective assessment of its generalization ability.^21^ From Table 3, the average AUC is 0.9123 (0.8350–0.9650), indicating good stability of our model (Figure 4).

### Feature selection and radiomics model construction

This study utilized a variety of statistical methods to analyze medical imaging data. Weighted feature ranking was conducted on all attributes, as feature extraction is pivotal in data processing. We adopted a multi‐stage feature extraction method.

The independent samples t‐test was used to assess relationships between features. Multivariate regression analysis evaluated the significance of each feature's regression coefficient. We organized the *p*‐values in ascending sequence, and the top 33% of features (around 633 features) underwent further scrutiny. This phase aimed to discard non‐influential features, minimizing data noise and redundancy.

To further optimize the feature subset, we applied MyLASSO regression to the remaining features for dimensionality reduction. By this method, we achieved dimensionality reduction and optimization of the data. We experimented with different values of λ ranging from $10^{-5}$ to $10^{2}$ and recorded the coefficients of the MyLASSO models corresponding to each value (Figure 3).

The optimal λ value was selected as $2.54\times 10^{-3}$, and the relationship graph (Figure 3) between MSE and λ demonstrated that this particular λ value indeed produced the best results. These coefficients represent the importance of features in the model. We identified 30 key features in our study.

To assess the performance of the selected features, we calculated the root mean squared error (RMSE), resulting in a value of 1.374.

$$RMSE=\sqrt{MSE}=\sqrt{\frac{1}{n}\sum_{i-1}^{n}{\left(y_{i}-{\hat{y}}_{i}\right)}^{2}}$$

This indicates that the extracted features demonstrate good predictive performance in current processing tasks.

### Analysis

Using the Extra Trees model, we re‐classified and predicted the 30 re‐extracted features, with results shown in Figure 4a–c. The results showcase strong classification performance: over 80% accuracy for GSL, GIST, GEP, and 100% for GML. However, the NET's smaller sample size limited its prediction accuracy to 75%. The precision‐recall (PR) curve of the model is depicted in Figure 4c, revealing impressive outcomes. Figure 4d–f contrasts the performance with the full feature set, whereas Figure 4a–c highlights superior results.

We also evaluated our prediction model with 10‐fold cross‐validation (Table 3) to gauge its tumor classification proficiency. Compared with Table 3, the model shows enhanced performance versus the full dataset. The elevated AUC implies the feature selection process effectively weeded out redundant or detrimental features. The model's average AUC of 0.9260 underscores its precision in distinguishing lesion types—essential for medical image classification. The model's mean values for sensitivity (0.7771) and specificity (0.7708) emphasize its capacity to correctly identify positive samples, crucial for early disease detection and minimizing false negatives.

In summary, our radiomics classification model, based on 10‐fold cross‐validation results, boasts high AUC, sensitivity, and specificity—affirming its accuracy, consistency, and vital role in tumor diagnosis and research.

## DISCUSSION

This study introduces an innovative radiomics model for identifying and classifying SMTs using EUS images. Analyzing EUS images from 144 patients, we extracted 1952 radiomic features. A rigorous process involving statistical testing and a custom LASSO regression identified 30 key features with significant discriminatory ability. These features underpinned a model with impressive diagnostic accuracy, indicated by an average AUC of 0.9260, and showed stability and calibration in validation cohorts.

Radiomics represents a significant step forward in diagnosing complex tumors, marking a first in SMT classification.^22^ It effectively captures comprehensive tumor data, offering insights into tumor characteristics previously unachievable with conventional methods.^23^ This research has vital clinical implications, particularly in preoperative diagnosis and treatment planning for SMTs, which, despite being mostly benign, can cause psychological stress and require surgical intervention, affecting recovery times.^24^

Our findings highlight radiomics' potential in SMT diagnosis automation, contrasting with recent studies like Binglan Zhang et al.’s work on using artificial intelligence for GIST diagnosis via EUS images, and another study's examination of artificial intelligence with contrast‐enhanced harmonic EUS for differentiating GISTs from leiomyomas.^25^, ^26^ Our approach encompasses a wider range of SMT classifications, demonstrating broader applicability and enhancing diagnostic methodologies in the field.

Compared to traditional diagnostic methods such as EUS fine‐needle aspiration, unroofing biopsy, or mucosal incision‐assisted biopsy, the methodology presented in this work, through detailed imaging feature extraction, achieves a non‐invasive and more comprehensive analysis of lesions. This represents a significant advancement over conventional invasive techniques, which are susceptible to sampling errors. The adoption of radiomics in medical diagnostic processes signifies a shift towards less invasive, more informative diagnostic practices, potentially revolutionizing the clinical decision‐making process for SMTs.

Nonetheless, our study is not devoid of limitations. The sample size, albeit sufficient for preliminary analysis, remains modest and warrants expansion in future research endeavors to enhance the model's generalizability and robustness. Additionally, the singular source of our data set—a lone medical center—could potentially introduce bias and limit the applicability of our findings across diverse clinical settings. Future studies are encouraged to embrace a multicentric approach, incorporating data from various geographical and demographic backgrounds to fortify the external validity of the model.

Radiomics is rapidly advancing, with improvements in imaging technologies and algorithms expected to enhance radiomic models' diagnostic accuracy and utility. Integrating radiomics with other diagnostic tools like MRI or CT could offer a more holistic approach to diagnosing SMTs.

In conclusion, our radiomics model signifies a significant step forward in the non‐invasive diagnosis of SMTs, offering a novel tool that potentially aids clinicians in the early detection, classification, and management of these tumors. Despite the highlighted limitations, this study paves the way for future research, emphasizing the need for larger, multicentric studies and the exploration of advanced imaging modalities to enhance the diagnostic landscape for SMTs.

## CONFLICT OF INTEREST STATEMENT

None.
